# Native Cyclodextrins as Complexation Agents for Pterostilbene: Complex Preparation and Characterization in Solution and in the Solid State

**DOI:** 10.3390/pharmaceutics14010008

**Published:** 2021-12-21

**Authors:** Laura Catenacci, Alexios I. Vicatos, Milena Sorrenti, Maria Cristina Bonferoni, Mino R. Caira

**Affiliations:** 1 Department of Drug Sciences, University of Pavia, Viale Taramelli 12, 27100 Pavia, Italy; laura.catenacci@unipv.it (L.C.); mariacristina.bonferoni@unipv.it (M.C.B.); 2 Department of Chemistry, University of Cape Town, Rondebosch 7701, South Africa; VCTALE001@myuct.ac.za

**Keywords:** pterostilbene, cyclodextrin, complexation, thermal analysis, X-ray diffraction

## Abstract

Pterostilbene (3,5-dimethoxy-4′-hydroxystilbene, PTB) is a natural dietary stilbene, occurring primarily in blueberries and Pterocarpus marsupium heartwood. The interest in this compound is related to its different biological and pharmacological properties, such as its antioxidant, anti-inflammatory, and anticarcinogenic activities and its capacity to reduce and regulate cholesterol and blood sugar levels. Nevertheless, its use in therapy is hindered by its low aqueous solubility; to overcome this limitation we studied the feasibility of the use of cyclodextrins (CDs) as solubility-enhancing agents. CDs are natural macrocyclic oligomers composed of α-d-glucose units linked by α-1,4 glycosidic bonds to form torus-shaped molecules, responsible for inclusion complex formation with organic molecules. In particular, the aim of this study was to evaluate the feasibility of complexation between PTB and native CDs using various preparative methods. The isolated solid products were characterized using differential scanning calorimetry (DSC), simultaneous thermogravimetric/DSC analysis (TGA/DSC), Fourier transform infrared (FT-IR) spectroscopy, and X-ray diffraction (XRD) on powder and single crystals. The results indicated little or no evidence of the affinity of PTB to complex with α-CD using the kneading method. However, with β-CD and γ-CD thermal analysis revealed an interaction which was also corroborated by FT-IR and ^1^H-NMR spectroscopy. With β-CD, a hydrated complex of PTB was isolated and its characterization by single-crystal XRD revealed, for the first time, the mode of inclusion of the PTB molecule in the cavity of a CD. To complement the solid-state data, liquid-phase studies were carried out to establish the effect of CDs on the aqueous solubility of PTB and to determine the complex stoichiometries and the association constants for complex formation. Phase-solubility studies showed A_L_-type profiles for α- and β-CD and a B_S_ profile for γ-CD, with K_1:1_ values of 1144, 4950, and 133 M^−1^ for α-CD·PTB, β-CD·PTB, and γ-CD·PTB, respectively. The stoichiometry of CD·PTB complexes, determined by Job’s method, revealed for each system a 1:1 molar ratio. The dissolution rate of PTB was approximately doubled just by employing simple physical mixtures, but the best performance was achieved by products obtained via kneading and co-precipitation, which effected the complete dissolution of PTB in 40 and 20 min for β-CD and γ-CD, respectively.

## 1. Introduction

The solubility enhancement of Biopharmaceutical Classification System (BCS) class II drugs represents an important challenge in preformulation studies and in pharmaceutical development, especially for oral delivery systems, as it influences systemic absorption and pharmacological responses [1]. To address this challenge, different approaches have been investigated, e.g., increasing the surface area of the drug sample by decreasing particle size [2], nanocrystallization [3,4], salt and cocrystal formation [5], solid dispersion technology [6], and the preparation of cyclodextrin (CD) inclusion complexes [7,8].

Pterostilbene (3,5-dimethoxy-4′-hydroxystilbene, PTB) is a natural phytoalexin occurring primarily in fruits, such as grapes and blueberries, and in Pterocarpus marsupium heartwood, belonging to Class II of the BCS [9]. PTB has attracted increasing interest, due to its therapeutic properties in several human diseases, which include neurological [10] and metabolic [11,12] disorders. In addition, it has been reported that it possesses antioxidant activity by scavenging reactive oxygen species (ROS) [13], anti-inflammatory activity by reducing the expression of some inflammatory mediators [14], as well as anti-cancer activities by inhibiting and/or preventing the activation of many signaling pathways involved in carcinogenesis [14,15]. Despite its various promising pharmacological properties, one of the principal factors that limits its utility in therapy is low aqueous solubility and its potential complexation with CDs was specifically investigated in the present study to address this shortcoming [16,17].

CDs are natural macrocyclic oligomers composed of α-d-glucose units linked by α-1,4 glycosidic bonds to form torus-shaped molecules. The formation of inclusion complexes with organic molecules, which can be included wholly or partially in the hydrophobic cavity, is favored by this conformation. CD complexation can be exploited, primarily to enhance the guest’s solubility but also to ameliorate other properties of the drug such as chemical stability, to prevent adverse side effects (e.g., gastrointestinal irritation), to mask unpleasant odors and flavors, and to prevent drug interactions with other components of the formulation (e.g., drug, active molecule and excipients) [18,19].

In the attempt to obtain CD∙PTB inclusion complexes, various methods were investigated, including simple physical mixing of the components, their co-precipitation, evaporation with microwave irradiation or rotavapor, kneading, and manual grinding.

The solid-state characterization of all isolated phases was performed using differential scanning calorimetry (DSC), thermogravimetric analysis (TGA), X-ray diffraction (XRD), and proton nuclear magnetic resonance (^1^H NMR) spectroscopy. As no previous structural data for CD∙PTB complexes appear in the Cambridge Crystallographic Database [20], single-crystal XRD was employed to obtain definitive structural details regarding the mode of inclusion of PTB in β-CD. Finally, to establish the real effect of CD complexation on the aqueous solubility of PTB and to calculate the association constants of the complexes, phase-solubility studies were carried out [21]. The stoichiometry of each CD-PTB inclusion complex formed was instead determined by Job’s method [22].

## 2. Materials and Methods

### 2.1. Materials

Pterostilbene (PTB) ≥ 99%, molecular weight: 256.3 g mol^−1^, was purchased from Mega Resveratrol Candlewood Stars Inc. (Danbury, CT, USA). α-CD 99%, molecular weight: 972.8 g mol^−1^, β-CD 98%, molecular weight: 1135 g mol^−1^, and γ-CD 98%, molecular weight: 1297.1 g mol^−1^ were purchased from Sigma Aldrich (St. Louis, MO, USA).

All other materials and solvents were of analytical-reagent grade. Phosphate buffer solution (PBS) with a pH of 7.4 was prepared according to the European Pharmacopeia monograph by dissolving potassium dihydrogen phosphate (0.2 M) and sodium hydroxide (0.1 M) in distilled water [23].

### 2.2. Methods

#### 2.2.1. Sample Preparation

Physical mixtures (PMs) were prepared by homogeneously mixing, in a Turbula mixer for 20 min, equimolar amounts of PTB with each CD, previously sieved to collect the particle size fraction < 250 μm. Each PM was then wetted in a mortar with ethanol and dried to a constant mass at 70 °C in an oven to obtain the kneaded product (KN); the procedure was repeated three times and the samples were then sieved through a 250 μm sieve.

Each PM was also subjected to manual trituration for 2 h using a mortar and pestle to obtain the ground product (GR).

In addition, PTB was dissolved in ethanol and the resultant solution was added dropwise to an aqueous solution of an equimolar amount of CD; the suspension was stirred for 6 h at 70 °C to obtain a clear solution and subsequently evaporated using a rotary evaporator under reduced pressure (RV) or microwave irradiation (MW) at 425 W (Pabisch CM-Aquatronic). The residues were gently ground in a mortar with a pestle and passed through a 250 μm sieve.

Co-precipitation products (CPs) were obtained by adding dropwise an equimolar amount of PTB dissolved in ethanol to a solution of each CD in water preheated to 70 °C. The resulting suspension was stirred for 1.5 h until a clear solution was obtained; the solution was cooled to room temperature and then stored in the refrigerator (4 °C) for 24 h. The resulting crystals were filtered and subsequently dried in a desiccator containing P_2_O_5_ under vacuum up to constant weight and then passed through a 250 μm sieve.

Single crystals of the hydrated β-CD∙PTB complex were isolated as follows. A 5 mg (0.02 mmol) sample of PTB was added to a 2.5 ml aqueous solution containing 45.4 mg (0.04 mmol) β-CD, which was preheated to 85 °C, while stirring vigorously with the exclusion of light. The PTB was added in miniscule increments, allowing dissolution to occur between each successive addition. The resultant solution was left to stir for 3 days and was subsequently filtered with a nylon 0.45 μm microfilter and placed in a Dewar flask to crystallize.

#### 2.2.2. Thermal Analysis

Differential Scanning Calorimetry (DSC) analyses were performed in triplicate in a DSC Mettler 821 STAR^e^ system (Mettler Toledo, Milan, Italy) equipped with a Module and an Intracooler device for subambient temperature analysis (Julabo FT 900, Julabo, Seelbach, Germany), previously calibrated using Indium as a reference. Samples of 2–4 mg (Mettler M3 Microbalance) were scanned from 30 to 350 °C in a sealed aluminum pan with a pierced lid (capacity 40 μl) with a heating rate of 10 K min^−1^ (nitrogen atmosphere, flux 50 ml min^−1^). An analogous empty pan was used as a reference.

Simultaneous Thermogravimetric Analysis (TGA) was performed in a TGA/DSC1 Mettler STAR^e^ system (Mettler Toledo, Milan, Italy), previously calibrated using Indium as a reference. Samples weighing 3–4 mg in alumina crucibles with lids were scanned from 30 to 350 °C (heating rate 10 K min^−1^) under nitrogen air atmosphere (flux 50 ml min^−1^). For analysis of the single-crystal samples of the CD∙PTB complex, DSC measurements were recorded on a DSC XP-10 instrument (THASS: Thermal Analysis & Surface Solutions GmbH, Friedberg, Germany) with a sample heating rate of 10 K min^−1^ and a dry N_2_ purge gas with a flux of 60 ml min^−1^. Thermogravimetry was performed under similar conditions on a TA-Q500 apparatus (Texas Instruments, Dallas, TX, USA). All measurements referred to above were carried out at least in triplicate.

#### 2.2.3. Fourier Transform Infrared Spectroscopy

Fourier transform infrared (FT-IR) spectra in the mid-IR range (650–4000 cm^−1^) were recorded using a PerkinElmer Spectrum One spectrophotometer (PerkinElmer, Monza, Italy) equipped with a single reflection ATR accessory (Pike MIRacle™ Technologies, Madison, WI, USA). Each sample, as such, was pressed onto an ATR crystal of ZnSe for the analysis. The spectra were collected in transmittance mode at least in triplicate (64 scans at a resolution of 4 cm^−1^).

#### 2.2.4. Nuclear Magnetic Resonance Spectroscopy

Proton Nuclear Magnetic Resonance (^1^H NMR) spectroscopy was used to determine the stoichiometry of the CD∙PTB complex. The host–guest ratio was determined by dissolving pure CD∙PTB complex crystals in deuterated dimethyl sulfoxide (DMSO-d_6_), recording the NMR spectrum of the resulting solution on a Varian-Gemini 300 spectrometer (Varian, Inc., Palo Alto, CA, USA) at 298 K, and analyzing the respective integrated signals.

#### 2.2.5. X-ray Diffraction

Powder X-ray diffraction (PXRD) patterns were collected on a Bruker D5005 powder diffractometer (Siemens, Germany) equipped with a θ–θ vertical goniometer and a Position Sensitive Detector (PSD, Braun, Garching, Germany). CuKα radiation (λ = 1.5418 Å) was employed with the generator settings 40 kV and 30 mA. Patterns were recorded in the step-scan mode (step: 0.015°, counting time: 0.5 s) in the angular range 5° < 2θ < 30° at room temperature. PXRD patterns of the products obtained with α-CD were recorded using a Bruker D8 Advance X-ray diffractometer (Bruker, Karlsruhe, Germany) using CuKα_1_-radiation (λ = 1.5406 Å) with the generator settings 30 kV and 40 mA (scan range 4.0–40.0°, step-size 0.05° s^−1^).

X-ray intensity data for the hydrated β-CD∙PTB complex were collected from a single crystal of the complex using a Bruker Apex II four-circle diffractometer and MoKα radiation (λ = 0.71073 Å). The crystal was maintained at 100(2) K in a stream of nitrogen vapor using an Oxford Cryostream cooler (Oxford Cryosystems Ltd, Oxford, UK). Direct phasing revealed the host molecule and the remaining atoms were located in successive difference Fourier maps. Structure solution and full-matrix least-squares refinement were performed with programs in the SHELX suite listed in the CIF file (see Supplementary Materials). While the host molecule presented a small level of disorder, the PTB molecule was found to be disordered over two distinct positions related by a pseudo-mirror plane. Most of the non-hydrogen atoms of the β-CD molecule were treated anisotropically, while the disordered guest atoms with fixed site-occupancy factors (s.o.f.s) of 0.5 were refined isotropically. To ensure that the molecules maintained reasonable geometries, numerous distance restraints were employed in the refinement. Eleven water oxygen atoms, disordered over 20 sites, were included in the model. Due to the majority of these atoms having s.o.f. <1, their H atoms were not discernible and hence were not included in the model. Full details of the structure determination and geometrical parameters for the complex are listed in the CIF file. A summary of the crystal and refinement data for the complex is provided [24].

#### 2.2.6. Phase-Solubility Analysis

Phase-solubility studies were performed according to the method described by Higuchi and Connors [25]. CDs were dissolved in Phosphate Buffered Saline (PBS) with a pH of 7.4 to yield solutions whose concentrations were in the range 0–100 × 10^−3^ M, for α-and γ-CD, and 0–10 × 10^−3^ M for β-CD. An excess of PTB (about 200 mg) was added to 10 ml of each CD solution and the solutions, protected from light, were shaken at 37 ± 1 °C for 48 h in a temperature-controlled water bath. Aliquots were withdrawn, subsequently filtered through 0.22 μm nitrocellulose filters, and diluted appropriately with the same medium. The concentration of PTB was determined using UV–Vis spectrophotometry at a wavelength of 306 nm, using a Lambda 20 UV–Vis spectrophotometer (PerkinElmer, Milan, Italy). The extinction coefficient of PTB was calculated by dissolving 1 mg in 10 ml of an ethanol solution. Absorbances were measured for different dilutions of this stock solution. From the slope of the concentration vs. absorbance curve, the extinction coefficient was calculated as 31,700 M^−1^cm^−1^ (see Supplementary Materials, Figure S1, calibration curve).

The solubility of PTB in the absence of CD (S_0_) was determined by preparing solutions containing an excess of PTB in the same medium and maintained in the same conditions described above until equilibrium was reached. All measurements were recorded in triplicate.

The apparent stability constant K_1:1_ of the complexes was estimated using the equation

(1) $$K_{1:1}=\frac{\mathrm{slope}}{S_{0}\;\left(1-\mathrm{slope}\right)}$$

where the slope was calculated from the linear portions of the phase-solubility diagrams and S_0_ was the drug solubility determined in the absence of CD.

#### 2.2.7. Job’s Method

Job’s method, commonly known as the continuous variation method, was employed to determine the stoichiometries of the CD∙PTB inclusion complexes [22]. Different aqueous solutions of PTB with each CD were prepared and maintained at 25 ± 1 °C, varying the mole fraction of the guest (X_PTB_) in the range between 0 and 1. Job plots were obtained by plotting ΔA, as the difference in the absorbance of PTB without and with CD, versus the PTB mole fraction. The absorbance values were determined using UV–Vis spectrophotometry at the PTB maximum wavelength of 306 nm. The stoichiometries of the inclusion complexes were obtained by taking the value of X_PTB_ at the maximum point on the curve. All measurements were recorded in triplicate.

#### 2.2.8. Dissolution Studies 

For the more promising CD-PTB systems, to evaluate the real solubilization effect of CD on PTB, dissolution studies were performed in a PBS solution with a pH of 7.4 at 37 ± 1 °C and a rotational speed of 100 rpm, according to the dispersed amount method. A sample of 5 mg of PTB or drug equivalent (sieved fraction < 250 μm) was added to 500 ml of dissolution medium. The studies were carried out with a paddle (Apparatus 1, Ph. Eur. [23]) using the instrument Electrolab Mod. TDT-082L (Electrolab, Bombay, India). Aliquots were withdrawn at prefixed time intervals, filtered using 0.22 mm nitrocellulose filters, and assayed for the concentration of PTB, as described above. Each test was performed in triplicate. For every time interval, the volume of sample withdrawn was replaced with an equal volume of fresh medium; a correction for the cumulative dilution was made, according to the following equation.

(2) $$Q_{t}=V_{R}C_{t}+\overset{t-1}{\underset{i=0}{\sum }}V_{S}C_{i}$$

Q*_t_* = PTB dissolved at time *t*

V_R_ = volume of dissolution medium

V_S_ = volume of aliquot withdrawn

C_i_ = PTB content at time i (i < *t*)

C*_t_* = PTB content at time *t*

## 3. Results

### 3.1. Thermal and FT-IR Characterization of the Commercial Product

Commercial PTB is a white crystalline powder with a thermal profile typical of an anhydrous sample.

**Figure 1:**
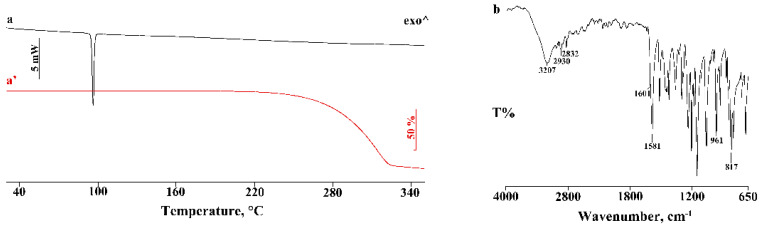
DSC (**a**) and TGA (**a’**) profiles and FT-IR spectrum (**b**) of commercial PTB. ^—represents the upward direction for exothermic (‘exo’) events.

In Figure 1, the DSC and TGA profiles (curves a’, a) are reported as well as the FT-IR spectrum (b) of the commercial product, for use as references for the pure compound in the study of the interaction products. In detail, the DSC curve showed a single endothermic effect at 95.9 ± 0.3 °C (*T_onset_* = 95.5 ± 0.2 °C; ΔH_m_ = 97 ± 2 J·g^−1^) due to the melting of the sample, corresponding to the PTB polymorph I described in the literature [26,27]. The TGA analysis of the sample confirmed the anhydrous nature of PTB, displaying a single mass loss of about 90% over 220 °C due to the thermal decomposition of the melted sample.

FT-IR analysis also confirmed that the commercial product is the more thermodynamically stable polymorph, showing the characteristic functional band at 3207 cm^−1^ due to the OH-stretching vibration, at 2930 and 2832 cm^−1^ due to the stretching of the aromatic groups, at 1601 cm^−1^ related to the stretching of the aromatic C–C double bond, at 1583 cm^−1^ related to the stretching of olefinic C=C, at 961 cm^−1^ due to the stretching of the trans-olefinic C–H, and at 817 cm^−1^ due to the vibrations of the C–H groups [28].

### 3.2. Thermal and FT-IR Characterization of the PTB-CD Binary Systems

**Figure 2:**
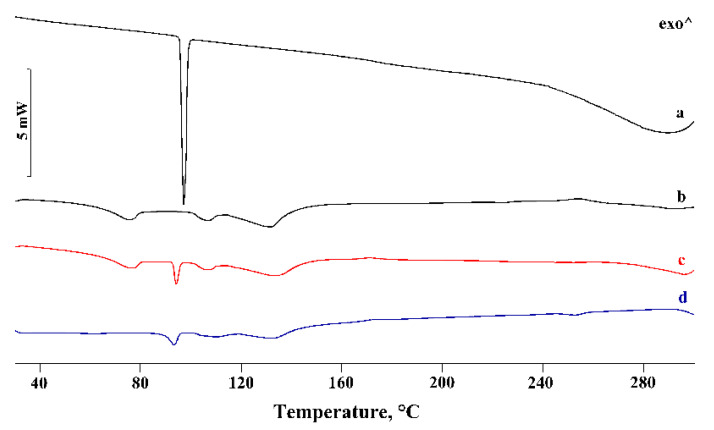
DSC profiles of commercial PTB (**a**), α-CD (**b**), their PM (**c**), and KN (**d**) products. ^—represents the upward direction for exothermic (‘exo’) events.

**Figure 3:**
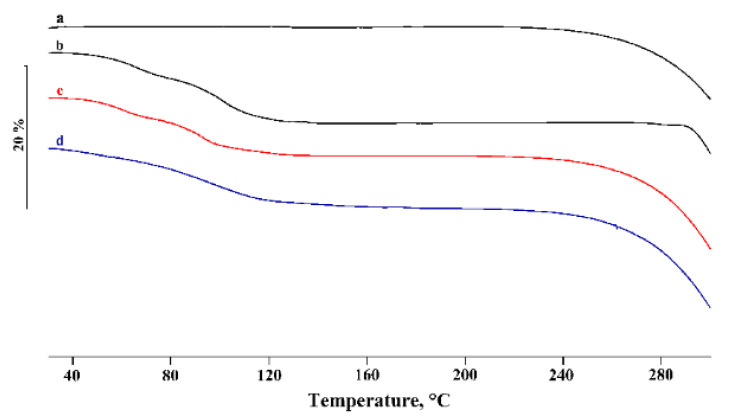
TGA profiles of commercial PTB (**a**), α-CD (**b**), their PM (**c**), and KN (**d**) products.

In Figure 2 and Figure 3, the DSC and TGA curves, respectively, of PTB, α-CD, their physical mixtures (PM), and the products obtained by kneading (KN) are compared.

The α-CD DSC curve showed three distinct effects in the temperature range of 30–150 °C, associated with the loss of water molecules from the CD cavity, with 10.3 ± 0.2% mass loss in the TGA profiles, in agreement with the theoretical value (10.0%). These effects were followed by melting at around 270 °C, before the decomposition confirmed by mass loss recorded in the TGA curve in the same temperature range. The PTB melting effect was revealed in the DSC curve of the PM (curve c) and treated via the kneading method (curve d). However, the reduction of the melting enthalpy values by 17% and 42% for the PM and KN products, respectively, relative to that of pure PTB, was probably due to a partial interaction and/or amorphization of the system. Similar results were obtained for the other samples of PM prepared by different methods (GR, RV, and MW), indicating little or no evidence of the affinity of PTB to complex with α-CD.

The FT-IR spectrum recorded on the PM appeared as the sum of the characteristic bands of both components, indicating its homogeneity. The absence of shifts in the wavenumbers for the treated systems confirmed that no interaction between PTB and α-CD was recorded, as already highlighted by the thermal data (see Supplementary Materials, Figure S2: FT-IR spectra). Supplementary Figure S3 shows the PXRD patterns of the PM, GR, KN, and CP products obtained for the α-CD-PTB system. GR resulted in an amorphous product with a few low-intensity peaks evident, all of which coincided with peaks in the PM. The patterns of both the KN and the CP products closely resembled that of the PM, with a small additional peak at 10.5° for the CP product and an intense peak at 11.0° for the KN product. The latter might represent the commencement of some form of interaction, but given the unusually long duration of the KN process (30 min), such a single peak is hardly evidence of a new crystalline phase. Thus, the PXRD results also support the conclusion of no significant evidence for complexation between α-CD and PTB drawn from the thermal and spectroscopic results above.

**Figure 4:**
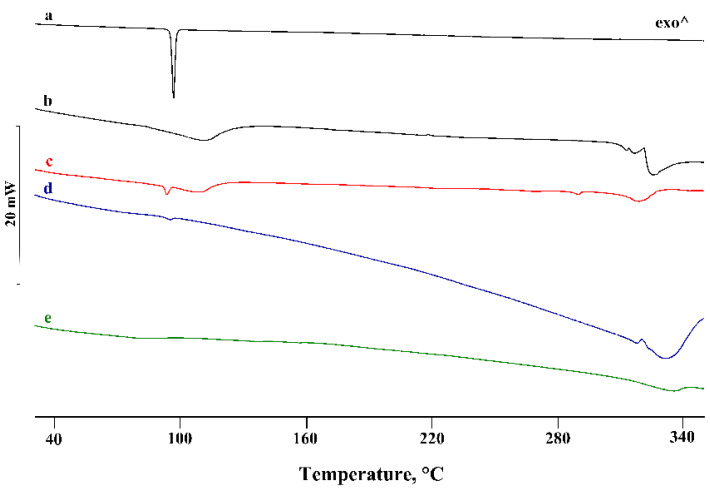
DSC profiles of commercial PTB (**a**), β-CD (**b**), their PM (**c**) and KN (**d**) and CP (**e**) products. ^—represents the upward direction for exothermic (‘exo’) events.

In the binary systems with the homologue β-CD, the PTB melting peak was still evident in the PM (Figure 4), with thermal and enthalpy parameters slightly lower than those of pure PTB (*T_peak_* = 92.8 ± 0.2 °C; ΔH_m_ = 71 ± 2 J g^−1^), probably due to CD impurity. Instead, in the treated PMs, a more significant reduction of PTB melting enthalpy was evident, indicating, for these binary systems, a solid-state interaction between the active molecules and the CD, resulting in a CP product for which the enthalpy value was about 85% lower than that of the pure PTB.

**Figure 5:**
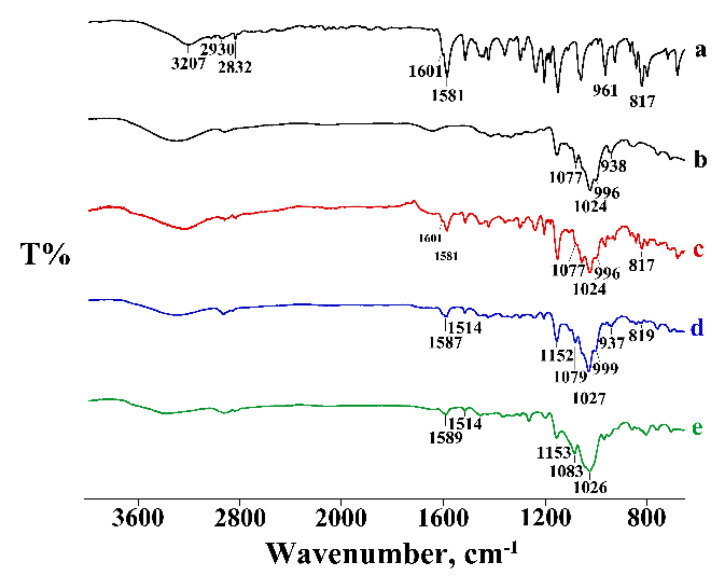
FT-IR spectra of commercial PTB (**a**), β-CD (**b**), their PM (**c**) and KN (**d**) and CP (**e**) products.

The thermal data were supported by the FT-IR analysis recorded on the same samples (Figure 5). While the PM spectrum manifested as a combination of the typical absorption bands of the two components, the spectra of the treated products showed a shift or a disappearance of some typical bands of PTB because of the host–guest interaction.

**Figure 6:**
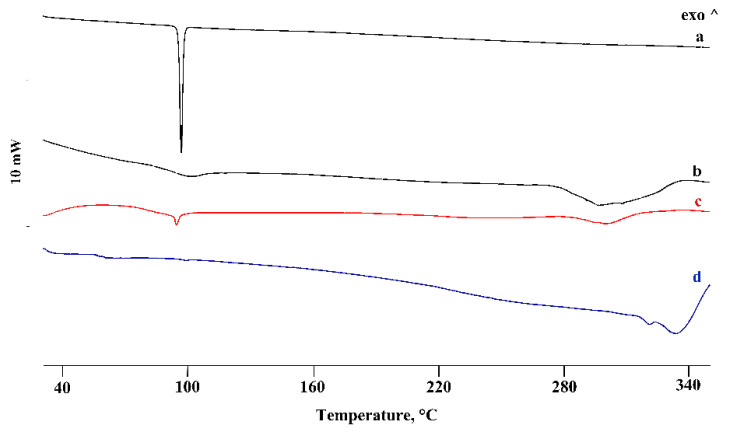
DSC profiles of commercial PTB (**a**), γ-CD (**b**), their PM (**c**) and KN (**d**) product. ^—represents the upward direction for exothermic (‘exo’) events.

For the last binary system with the higher homologue γ-CD, analogous deductions can be made regarding the PM, while for the treated systems the complete disappearance of the PTB melting peak indicated the inclusion of PTB in the hydrophobic CD cavity. As examples, Figure 6 shows the DSC curves of the binary systems treated via the KN method.

**Figure 7:**
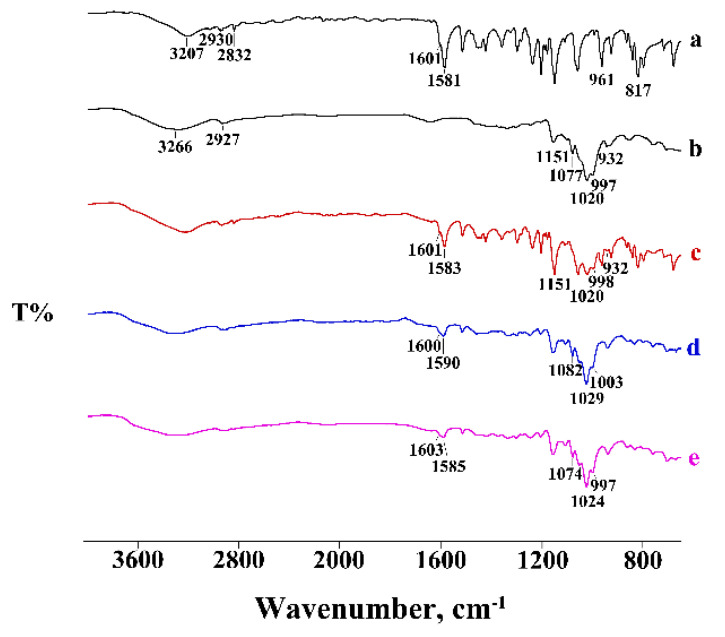
FT-IR spectra of commercial PTB (**a**), γ-CD (**b**), their PM (**c**) and KN (**d**) and RV (**e**) products.

In the case of the γ-CD-PTB system, the thermal data were also supported by FT-IR analysis; the bands of the two components, still present at the same wavenumber in the PM spectrum, shifted or did not appear in the spectra of the treated samples, confirming the formation of a complex (Figure 7).

### 3.3. X-ray Structure of the Hydrated β-CD Complex of PTB

Prior to the X-ray analysis, it was necessary to determine the composition of the ternary complex. Thermal analysis of single crystals (see Supplementary Materials, Figure S4: TGA and DSC curves) yielded a 13.1 ± 0.4% (*n* = 3) mass loss due to crystal dehydration. For a 1:1 β-CD–PTB stoichiometry, this mass loss corresponds to 11.6 ± 0.4 water molecules per β-CD molecule. On refinement of the crystal structure, the sum of the site-occupancy factors (s.o.f.s) of ordered and disordered water oxygen atoms located in difference Fourier syntheses was 11.3 per host molecule, in good agreement with the TGA value. However, for a more rigorous confirmation, the water oxygen atoms were temporarily deleted from the X-ray model and the PLATON Squeeze routine [29] was employed to calculate the total electron count in the resulting crystal voids. This calculation indicated a void volume of 1216 Å^3^ (i.e., 16% of the unit-cell volume) containing a total of 439 electrons, the latter value thus corresponding to 43.9 water molecules per unit cell, and hence (with 4 host molecules per unit cell) 11.0 H_2_O molecules per β-CD molecule. These results are consistent, strongly supporting the stated composition of the dimeric complex based on the TGA data, namely 2(β-CD) 2(PTB) 23.2H_2_O.

**Figure 8:**
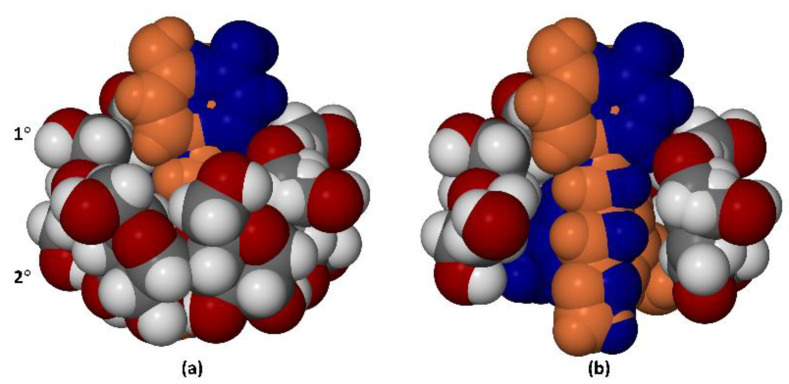
The mode of inclusion of the two disordered PTB components (orange and blue) in the β-CD molecule (**a**), and a cutaway view (**b**) showing the pseudo-mirror relationship between the ‘overlapping’ PTB components. The symbols 1° and 2° indicate the primary (narrow) rim of the host molecule and the secondary (wider) rim, respectively. The phenolic rings of PTB are located near the primary rim of the host and the 3,5-dimethoxyphenyl rings are near the secondary rim.

Figure 8 shows the structure of the monomeric unit in the crystal of the dimeric complex. While the extent of disorder of the host molecule is very small, involving only one of the seven hydroxymethyl groups on the primary rim, the PTB guest molecule is fully disordered, occupying two distinct sites in close proximity with site-occupancy factors (s.o.f.s) of 0.5 each and spanning the host cavity with some degree of guest protrusion from both the primary and secondary rims.

**Figure 9:**
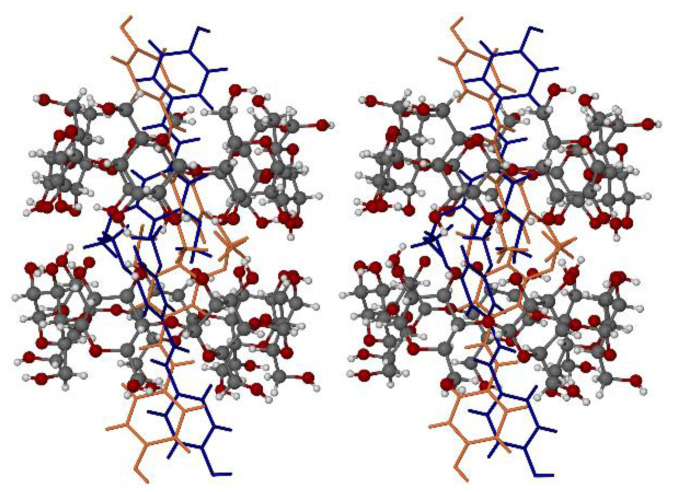
A stereoscopic view of the dimeric complex unit 2(β-CD) 2(PTB). The C_2_-axis (not shown explicitly) is horizontal and runs through the dimer interface.

The conformation of the β-CD molecule is stabilized by several intramolecular O-H⋯O hydrogen bonds that reinforce the secondary rim (see Supplementary Materials, Figure S5, stereoscopic view) as well as intermolecular O-H⋯O hydrogen bonds between the abutting secondary rims of the dimer (not shown). A stereoscopic view of the full dimeric unit is shown below (Figure 9) This unit comprises two monomeric units which are related by a crystallographic C_2_-axis parallel to the crystal b-axis. The mode of guest inclusion within the dimeric unit features the location of the lipophilic methoxy groups of PTB at the center of the dimeric β-CD ‘cage’ and protrusion of the phenolic residues from the primary rims into a hydrophilic zone where their interaction with a network of H-bonded water molecules occurs. As such, this structure serves as a useful model for future studies of CD-PTB interaction in the solid state and may also be relevant in the solution state. Lists of bond lengths, bond angles and torsion angles appear in the CIF file. The guest components display small but significant deviations from planarity as a result of their accommodation within the host dimer.

The computed PXRD pattern of the hydrated β-CD PTB complex is shown in Supplementary Materials, Figure S6, for reference. The unit-cell dimensions of the complex are very similar to those of the hydrated β-CD complex of 4,7-dimethylcoumarin (CSD refcode MASBAJ) and that of 4-allyl-2-methoxyphenol (CSD refcode TEZZUV) [20], both of which also crystallize in the monoclinic space group C_2_. Furthermore, the three complexes have isostructural host packing arrangements. (See Supplementary Figure S7 showing the computed PXRD traces based on only the host atom coordinates of the PTB complex and those of e.g., MASBAJ as representative example). However, these complexes form a small subgroup with a common host packing that differs somewhat from that of the more populous group of β-CD complexes crystallizing in C_2_. Their unit-cell volumes are also ~13% larger on average than those of the more common variety. A representative example of the latter group is a β-CD complex containing the guest methylparaben (CSD refcode AJUVEG). Supplementary Figure S7 shows the resulting difference in the PXRD pattern based on the host structural arrangement of AJUVEG.

The packing arrangement in the complex crystal 2(β-CD) 2(PTB) 23.2H_2_O is shown in Supplementary Figure S8. Dimeric complex units are stacked to form columns that are parallel to the crystal c-axis. The interstitial spaces between the columns contain water molecules which engage in a complex network of O-H⋯O hydrogen bonds that include water–water, water–host and water–guest interactions, which are responsible for maintaining the crystallinity of the ternary system.

### 3.4. Phase-Solubility Analysis

**Figure 10:**
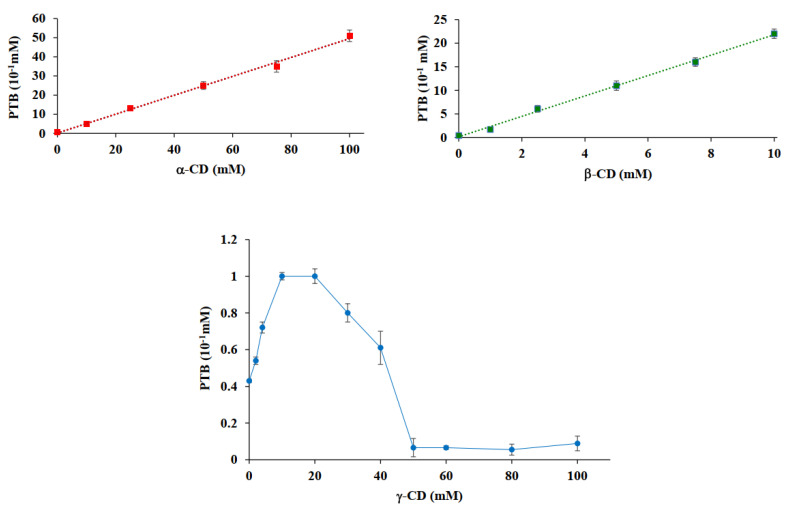
Phase-solubility profiles of PTB as a function of [α-CD] (orange), [β-CD] (green), and [γ-CD] (blue). Results were expressed as means ± standard deviations.

In Figure 10, phase-solubility profiles obtained by plotting the concentration of PTB against the concentrations of the natural CD employed in the experiment are presented. According to Higuchi and Connors [25], such diagrams can be classified into two types, the A-type corresponding to the formation of a soluble complex and the B-type corresponding to the formation of an insoluble complex.

The phase-solubility profiles resulting from the use of α-CD and β-CD were of type A_L_, with an increase in the apparent solubility of PTB due to soluble complex formation. In detail, these hosts produced a guest solubility enhancement of 100-fold and 40-fold for α-CD and β-CD, respectively, over the indicated concentration range. The results for the experiments with γ-CD revealed a B_S_ profile; the precipitation of an insoluble complex or aggregated CD particulates was physically observed during the experiments and the concentration of PTB in solution increased 2-fold at the beginning of the experiments but decreased with a further increase in CD concentration. Similar behavior was already observed with the homologous resveratrol, as reported in a previous study [7]. The 1:1 apparent stability constants, K_1:1_, were 1144, 4950, and 133 M^−1^ for α-CD·PTB, β-CD·PTB, and γ-CD·PTB, respectively. For the calculation of the K_1:1_ constant for complexation with γ-CD, only the initial slope up to 10 mM was used and the obtained value indicated a relatively weak interaction between PTB and this CD.

### 3.5. Analyses Based on Job’s Method

**Figure 11:**
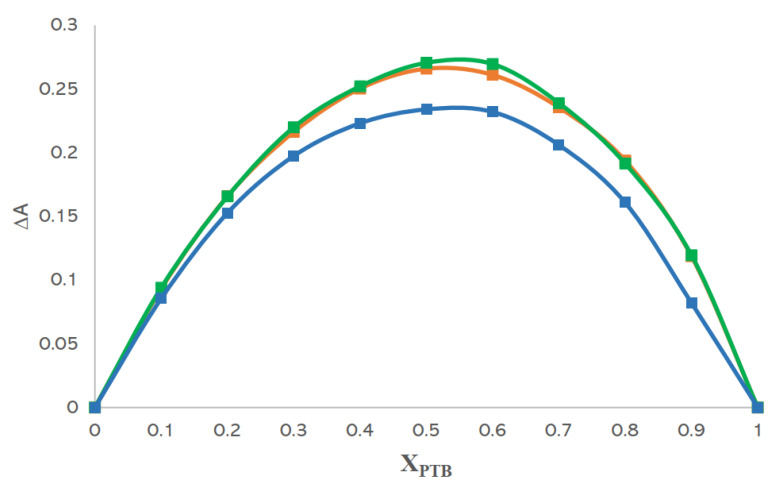
Job plots of α-CD·PTB (orange), β-CD·PTB (green), and γ-CD·PTB (blue) complexes.

The equimolar stoichiometry of CD·PTB inclusion complexes was confirmed using Job’s method. Job plots were obtained by plotting ΔA, as the difference in absorbance of PTB without and with CD, versus the PTB mole fraction. The obtained results showed a maximum in each point corresponding to X_PTB_ ∽ 0.5, indicating a 1:1 stoichiometry (Figure 11).

### 3.6. Dissolution Studies

**Figure 12:**
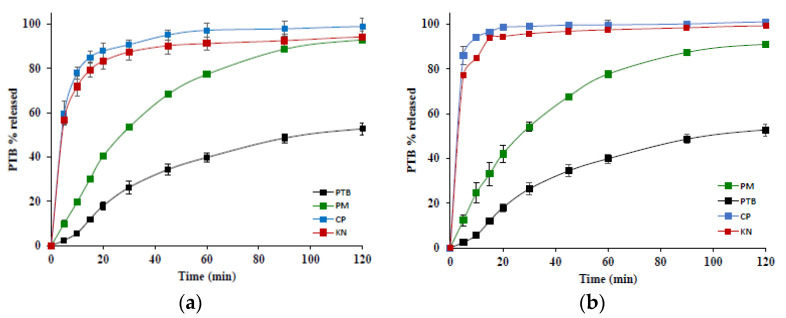
Dissolution profiles of PTB alone (black) and its PM (green), and the CP (blue) and KN (red) products with β-CD (**a**), and γ-CD (**b**). Results were expressed as means ± standard deviations.

The two binary systems with β- and γ-CD were also evaluated in terms of their ability to enhance the PTB dissolution properties. In Figure 12, the dissolution profiles of the physical mixtures, before and after treatment, were compared to those of PTB alone.

For both systems the simple PM effected an increase in the dissolution rate of PTB of about 2-fold. The best performance was displayed by the treated products, confirming the greater extent of the interaction obtained by these preparative methods as already revealed by the solid-state characterization results. The total amount of PTB dissolved was attained in 40 and 20 min for β-CD and γ-CD, respectively. The higher efficiency of the γ-CD-PTB system is probably attributable to the higher solubility of this CD than its lower homologue.

Attempts to obtain single crystals of the γ-CD complex of PTB for full structural elucidation were not successful due to its insolubility. However, the pure inclusion complex was isolated by a special procedure and the host–guest ratio was found to be 1:1 from the ^1^H NMR spectrum recorded in DMSO-d_6_ (see Supplementary Materials, Figure S9 and the description of the complex preparation). The PXRD pattern of this material was also recorded (Supplementary Figure S10) and was found to match the common reference pattern for the well-known isostructural series of tetragonal γ-CD inclusion complexes [30]. From this match, it was deduced that the γ-CD·PTB complex crystallizes in the tetragonal space group P42_1_2 with the unit-cell dimensions of a ~ 23.8 Å, c ~ 24.0 Å, and hence the extended host packing arrangement is that of the well-known isostructural series. Further analyses (e.g., TGA, DSC) were performed but are not recorded here, given the proven insolubility of the γ-CD·PTB complex in water, which renders it less relevant than, e.g., the β-CD·PTB complex in the pharmaceutical context.

## 4. Discussion

The present investigation focused on the feasibility of including the antioxidant PTB in the native cyclodextrins α-, β-, and γ-CD, the results indicating little or no evidence of the affinity of PTB to complex with α-CD. However, with the higher homologues β-CD and γ-CD a definitive indication of complexation was first evident from thermal analysis and subsequently corroborated by FT-IR and ^1^H-NMR spectroscopy, as well as PXRD and single-crystal X-ray diffraction methods.

Refinement of the crystal structure of the hydrated β-CD·PTB complex presented challenges due to the disorder of both the guest molecules and water molecules, and thus several models were developed before arriving at the final one reported here. The utility of the SQUEEZE routine in the program PLATON in obtaining a reliable electron count for accurate crystal water content estimation has been highlighted above. However, it should be mentioned that its application in this case was somewhat unusual in that, following temporary deletion of the assigned water oxygen atoms for the purpose of their electron count estimation, these atoms were subsequently restored to the model, based on the resulting agreement between calculated and experimental water content. Such an ‘atomistic’ model of solvent disorder is considered superior to one for which the full SQUEEZE procedure is followed to calculate the solvent contribution to the structure factors by back-Fourier transformation of the electron density difference detected in the crystal voids [29]. The X-ray structure of the hydrated β-CD·PTB complex reveals for the first time the mode of inclusion of the antioxidant in a cyclodextrin in the solid state.

To confirm the propensity of CDs as solubility enhancers, phase-solubility studies were carried out showing A_L_-type profiles for α- and β-CD and a B_S_ profile for γ-CD. The stoichiometry of CD·PTB complexes determined by Job’s method revealed for each system a 1:1 molar ratio.

The dissolution studies were carried out for the binary systems of PTB with β- and γ-CD which had shown more extensive interaction in the solid-state studies. The dissolution rate of PTB was increased just by simple PMs but the best performance was achieved by KN and CP products.

## 5. Conclusions

To increase the solubility of PTB, a class II substance according to the BCS classification, the feasibility of obtaining inclusion complexes with native CDs was investigated in this study. Both β-CD and γ-CD, which we had proven by X-ray methods to form crystalline inclusion complexes with PTB, were also chosen as hosts for phase solubility and dissolution rate studies, the latter revealing very significant increases in PTB solubility and dissolution rate. The best dissolution performance was achieved with CD-PTB systems containing the more soluble γ-CD. The hydrated β-CD·PTB complex was characterized by single-crystal XRD which revealed for the first time the mode of inclusion of this natural antioxidant in a cyclodextrin.

## Data Availability

CCDC 2123743 contains the supplementary crystallographic data for the crystal structure of the hydrated β-CD complex of PTB. These data can be obtained free of charge via http://www.ccdc.cam.ac.uk/conts/retrieving.html, accessed on 16 December 2021 (or from the CCDC, 12 Union Road, Cambridge CB2 1EZ, UK; Fax: +44-1223-336-033; E-mail:deposit@ccdc.cam.ac.uk).

## Supplementary Materials

The following are available online at https://www.mdpi.com/article/10.3390/pharmaceutics14010008/s1, Figure S1: Calibration curve of PTB, Figure S2: FT-IR spectra of α-CD-PTB PM (a) and KN (b) and MW (c) and RV (d) products, Figure S3: PXRD patterns for the PM, GR, KN and CP products obtained with α-CD and PTB, Figure S4: TGA and DSC curves for the complex 2(β-CD) 2(PTB) 23.2H_2_O, Figure S5: Stereoscopic view of the β-CD·PTB monomer showing the intramolecular hydrogen bonds on the secondary rim and the disordered guest components., Figure S6: PXRD pattern of 2(β-CD) 2(PTB) 23.2H_2_O computed from the refined single crystal X-ray model. (CuKα-radiation, λ = 1.5406 Å)., Figure S7: Computed PXRD patterns based solely on the respective host molecule arrangements in the crystals of complexes MASBAJ, 2(β-CD) 2(PTB) 23.2H_2_O, and AJUVEG. (CuKα-radiation, λ = 1.5406 Å). The high level of isostructurality of the host assemblies in MASBAJ and the reported β-CD·PTB complex is evident, Figure S8: The [001] projection of the crystal structure of 2(β-CD) 2(PTB) 23.2H_2_O, highlighting the C-centred arrangement of the complex units. Infinite columns of dimeric complex units parallel to the c-axis (view direction) are separated by interstices containing the water molecules (whose oxygen atoms are represented by isolated red spheres), Figure S9: The ^1^H NMR spectrum of the γ-CD·PTB inclusion complex prepared by the method described below, Figure S10: The experimental PXRD pattern of a sample of the γ-CD·PTB complex.
