# Supplementary material for: Native Cyclodextrins as Complexation Agents for Pterostilbene: Complex Preparation and Characterization in Solution and in the Solid State
Source: Pharmaceutics. 2021 Dec 21;14(1):8. doi: 10.3390/pharmaceutics14010008 (PMC8777607; doi:10.3390/pharmaceutics14010008)
Supplement: Supplementary file 1 [file pharmaceutics-14-00008-s001.zip › pharmaceutics-1510409-supplementary.pdf]

pharmaceutics

Supplementary Materials: Native Cyclodextrins as Complexation Agents for Pterostilbene: Complex Preparation and Characterization in Solution and in the Solid State

Laura Catenacci, Alexios I. Vicatos, Milena Sorrenti, Maria Cristina Bonferoni and Mino R. Caira

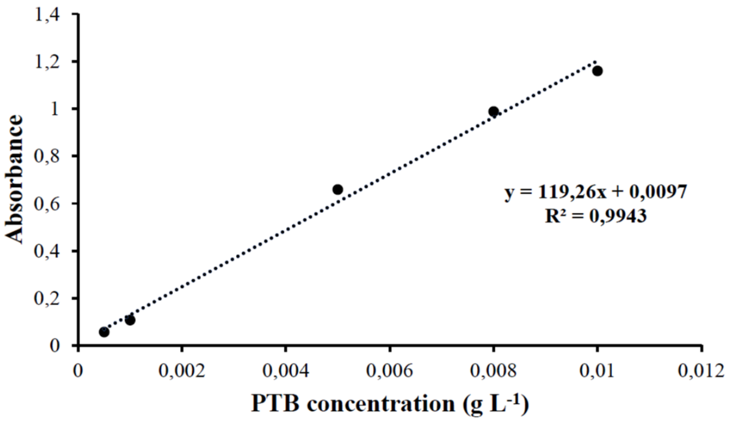

Figure S1. Calibration curve of PTB.

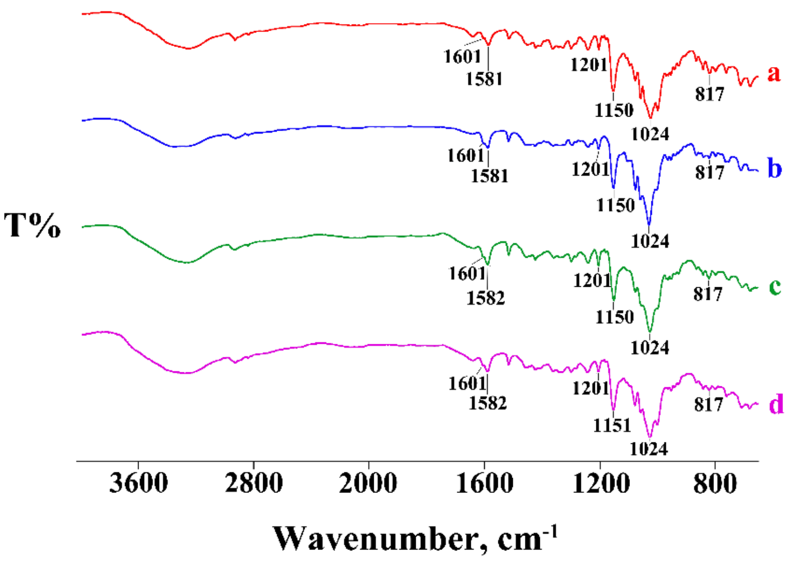

Figure S2. FT-IR spectra of α-CD-PTB PM (a) and KN (b) and MW (c) and RV (d) products.

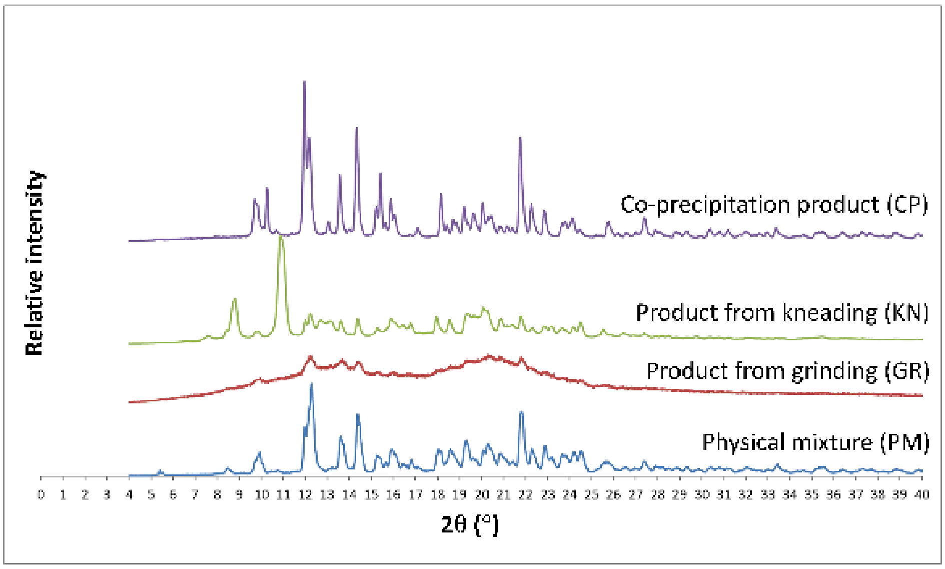

Figure S3. PXRD patterns for the PM, GR, KN and CP products obtained with α-CD and PTB.

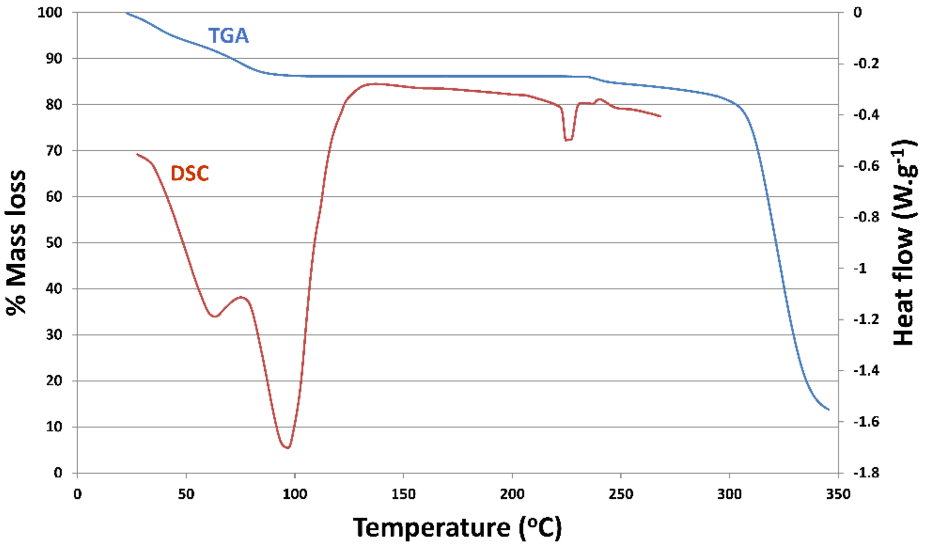

Figure S4. TGA and DSC curves for the complex 2(β-CD) 2(PTB) $23.2H_2O$.

The DSC trace displayed two thermal events over the recorded temperature range both of which correlated with the TGA analysis. The first thermal event was evident from the two merged, broad endothermic peaks with onset temperature 40.0 ± 2.7 °C (main peak at 98.3 ± 1.4 °C), representing two-step dehydration of the complex. The second thermal event is the commencement of partial loss of pterostilbene from the dehydrated inclusion complex, characterized by a small sharp endothermic peak with onset temperature 223.0 ± 0.2 °C (peak at 225.0 ± 0.3 °C). The thermal events depicted in both the DSC and TGA traces complement and reaffirm one another, even though there are variations between the TGA and DSC onset values of the same thermal event. This discrepancy is due to the fact that the analyses were conducted on different instruments.

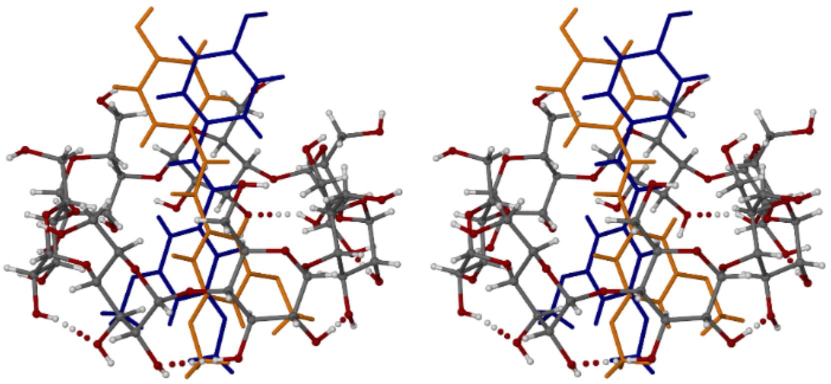

Figure S5. Stereoscopic view of the β-CD·PTB monomer showing the intramolecular hydrogen bonds on the secondary rim and the disordered guest components.

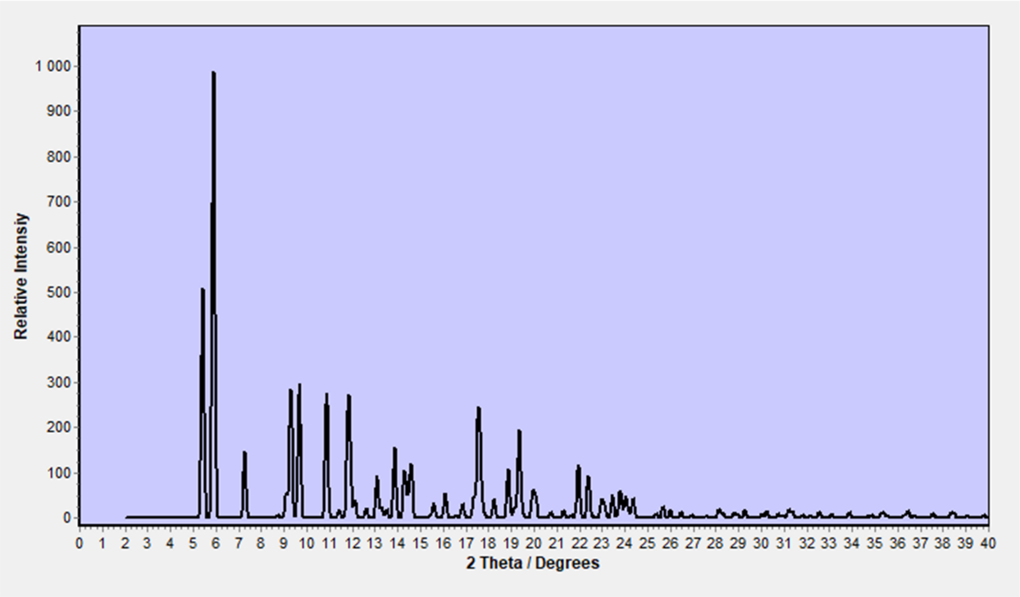

Figure S6. PXRD pattern of 2(β-CD) 2(PTB) 23.2H_2O computed from the refined single crystal X-ray model. (CuKα-radiation, λ = 1.5406 Å).

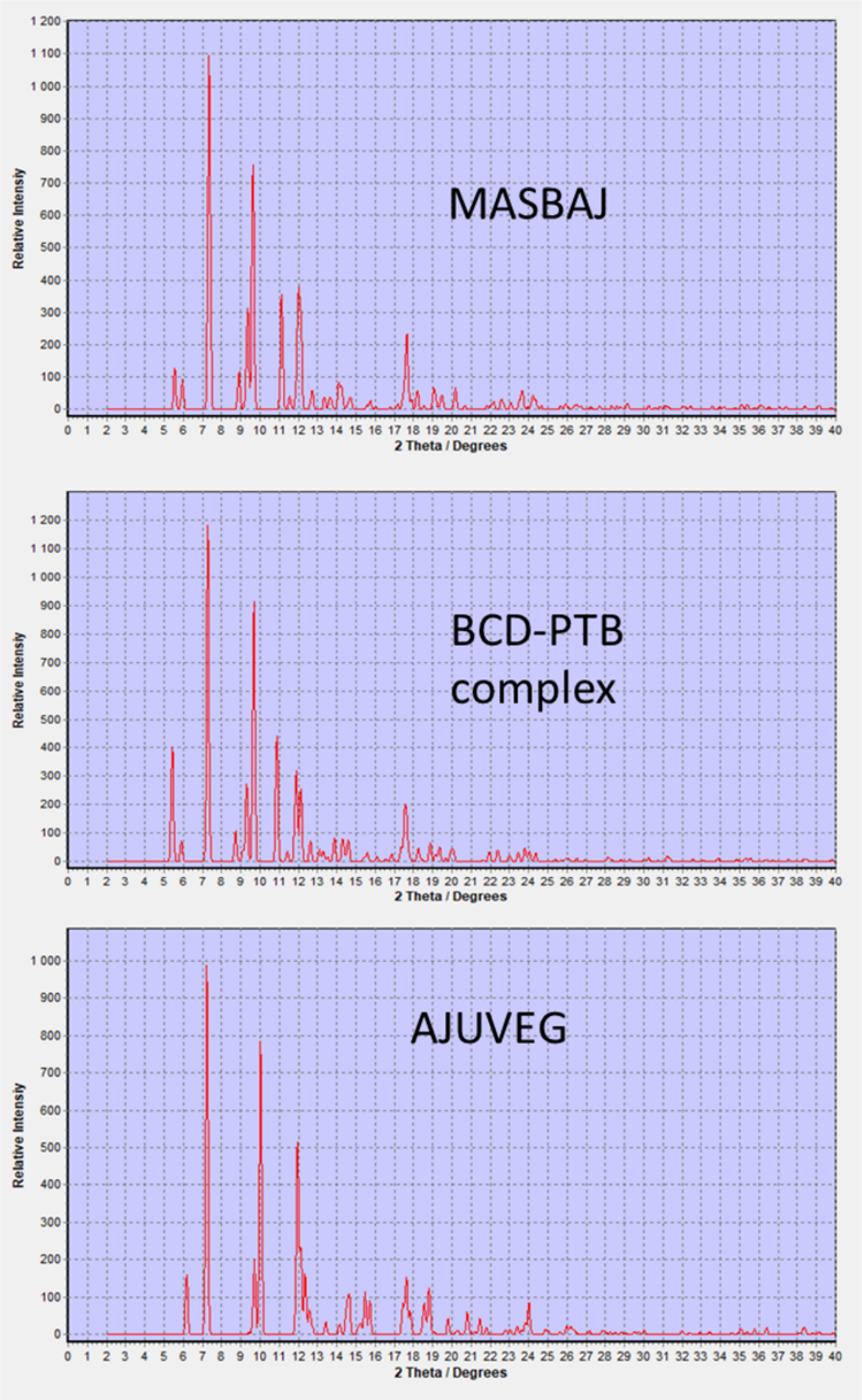

Figure S7. Computed PXRD patterns based solely on the respective *host molecule arrangements* in the crystals of complexes MASBAJ, 2(β-CD) 2(PTB) 23.2H_2O, and AJUVEG. (CuKα-radiation, λ = 1.5406 Å). The high level of isostructurality of the host assemblies in MASBAJ and the reported β-CD·PTB complex is evident.

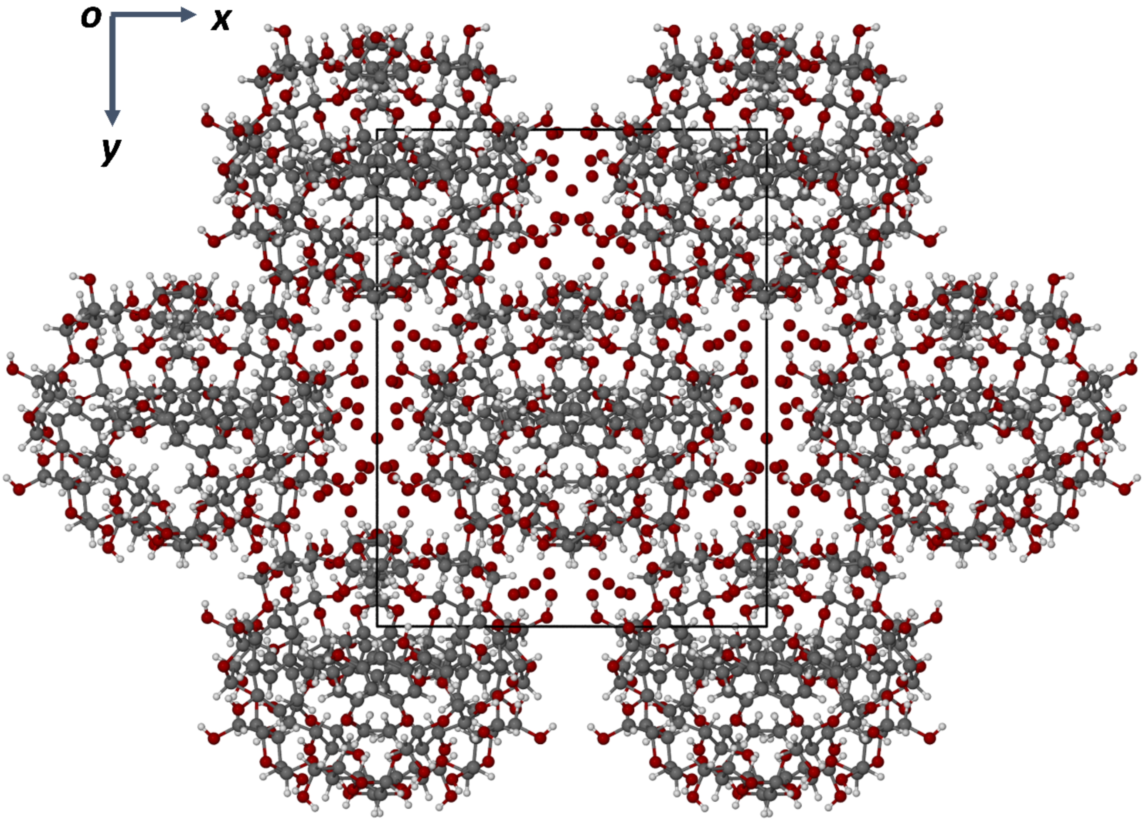

Figure S8. The [001] projection of the crystal structure of 2(β-CD) 2(PTB) 23.2H2O, highlighting the C-centred arrangement of the complex units. Infinite columns of dimeric complex units parallel to the c-axis (view direction) are separated by interstices containing the water molecules (whose oxygen atoms are represented by isolated red spheres).

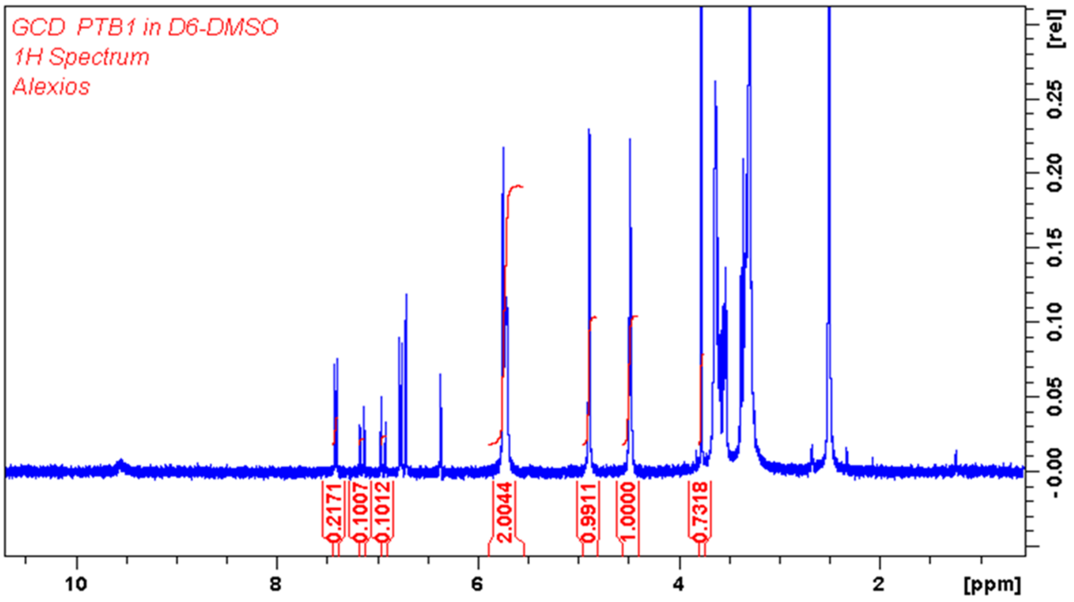

Figure S9. The ^{1}H NMR spectrum of the γ-CD·PTB inclusion complex prepared by the method described below.

Preparation of a pure sample of the γ-CD·PTB complex

10.0 mg (0.039 mmol) of pterostilbene and 50.6 mg (0.039 mmol) of γ-CD was kneaded for 40 min with 1 mL of water. The product was subsequently added to a vial containing 10 mL of water at 60 °C while stirring. It is important to note that the complex is the insoluble species (the suspended material) in the liquid medium. In order to ensure complex purity, a slight excess of γ-CD was added to the contents of the vial to ensure that all the insoluble pterostilbene had reacted. Thereafter, the solution with the suspension was slowly filtered with a nylon 0.45 µm microfilter and the filtrate was subsequently washed with water by slowly injecting 1 mL of water into the filter with a syringe. This ensured that all the excess γ-CD had been removed. The γ-CD·PTB complex filtrate was harvested by clamping the nylon filter horizontally, cutting it open with a sharp knife and leaving it to dry overnight. The product (which is white and translucent) could then be scraped very carefully off the membrane of the filter. In order to prove that the collected filtrate was an inclusion complex with a 1:1 host-guest molar ratio, a mixture of the host and guest with a 2.5:1 host-guest ratio was kneaded and prepared using the method described above. This product was used for the 1H NMR analysis, which yielded 1:1 host-guest stoichiometry.

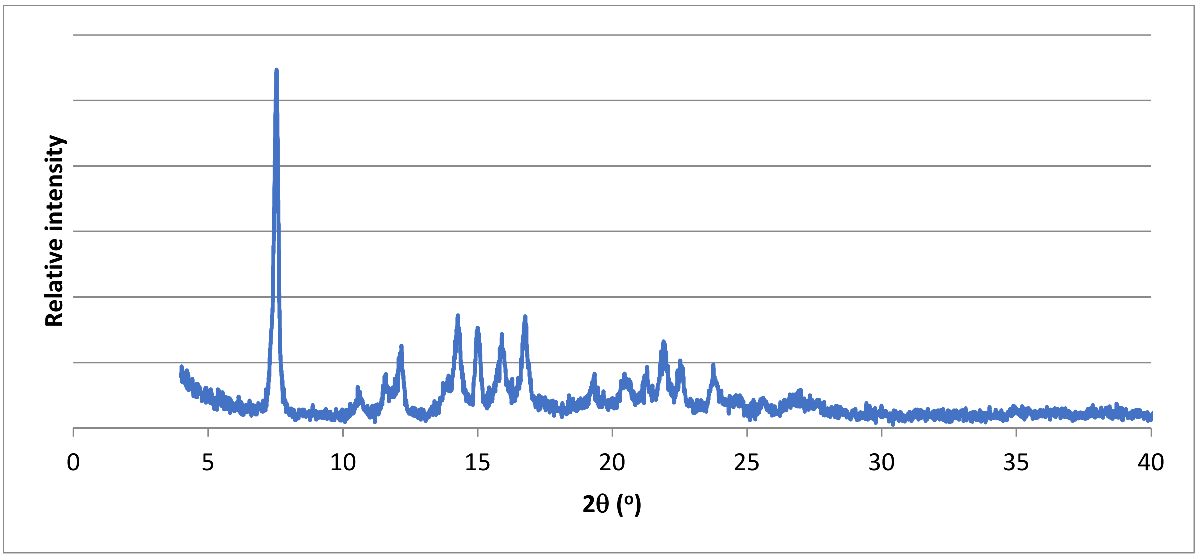

Figure S10. The experimental PXRD pattern of a sample of the γ-CD·PTB complex.
